# Complete Mitochondrial DNA Genome of Nine Species of Sharks and Rays and Their Phylogenetic Placement among Modern Elasmobranchs

**DOI:** 10.3390/genes12030324

**Published:** 2021-02-24

**Authors:** Vasiliki Kousteni, Sofia Mazzoleni, Katerina Vasileiadou, Michail Rovatsos

**Affiliations:** 1 Department of Ecology, Faculty of Science, Charles University, Viničná 7, 12800 Prague, Czech Republic; kousteni@inale.gr (V.K.); sofia.mazzoleni@natur.cuni.cz (S.M.); kvasileiadou@hcmr.gr (K.V.); 2 Institute of Marine Biology, Biotechnology and Aquaculture, Hellenic Centre for Marine Research, Thalassocosmos, P.O. Box 2214, 71003 Heraklion, Crete, Greece

**Keywords:** Chondrichthyes, complete mitogenome, phylogeny, rays, sharks, systematics

## Abstract

Chondrichthyes occupy a key position in the phylogeny of vertebrates. The complete sequence of the mitochondrial genome (mitogenome) of four species of sharks and five species of rays was obtained by whole genome sequencing (DNA-seq) in the Illumina HiSeq2500 platform. The arrangement and features of the genes in the assembled mitogenomes were identical to those found in vertebrates. Both Maximum Likelihood (ML) and Bayesian Inference (BI) analyses were used to reconstruct the phylogenetic relationships among 172 species (including 163 mitogenomes retrieved from GenBank) based on the concatenated dataset of 13 individual protein coding genes. Both ML and BI analyses did not support the “Hypnosqualea” hypothesis and confirmed the monophyly of sharks and rays. The broad notion in shark phylogeny, namely the division of sharks into Galeomorphii and Squalomorphii and the monophyly of the eight shark orders, was also supported. The phylogenetic placement of all nine species sequenced in this study produced high statistical support values. The present study expands our knowledge on the systematics, genetic differentiation, and conservation genetics of the species studied, and contributes to our understanding of the evolutionary history of Chondrichthyes.

## 1. Introduction

Cartilaginous fish (Class Chondrichthyes) consist a group of vertebrates that demonstrate an old radiation, dating back about 400 million years [1,2]. This lineage has survived four mass extinction events [1] and most present-day taxa derive from Mesozoic forms [3]. The evolutionary success of Chondrichthyes is partly due to the efficiency of their *K*-selective reproductive traits [4], such as large body size, slow growth rate, late maturity, low fecundity and large offspring size [5,6,7]. The class Chondrichthyes, which comprises the most diverse group of large predatory animals, currently includes 14 orders, 60 families and 198 genera with approximately 1200 species. It is composed of two subclasses, the Holocephali (chimaeras) including one superorder (Holocephalimorpha with 49 species), and Elasmobranchii including three superorders: Galeomorphii and Squalomorphii with 347 and 157 shark species, respectively, and Batoidea with 639 species of rays, stingrays, skates and sawfishes [8,9,10].

Chondrichthyes play an important ecological role, most notably functioning as either top predators with top-down control on the size and dynamics of many species [11] or mesopredators in the marine food webs [12,13] by linking different trophic levels in the marine ecosystems and contributing to system dynamics and stability [14]. Furthermore, apart from providing an important perspective to interpreting functional and life-history evolution as being the sister group to all other extant jawed vertebrates (Gnathostomata) [15], they exhibit a genomic architecture that is likely closer to the ancestral vertebrate condition compared to teleosts [16]. Their commercial value, especially of their meat, fin and liver is increasing as targeted teleost fish become less accessible [17,18]. As a result, overfishing has profoundly altered shark and ray populations [19,20,21] and several species are facing a two-fold higher extinction risk compared to finfish [22]. The International Union for the Conservation of Nature (IUCN) has assessed the conservation status of 604 out of 1192 taxonomically valid species with 179 chondrichthyans categorized as threatened (Vulnerable—VU; Endangered—EN; Critically Endangered—CR) [2]. Due to the fact that almost 1/3 of the assessed species face extinction risk [2,23] and further 63 Data Deficient (DD) species were predicted to be threatened based on correlates of IUCN threat status, chondrichthyans comprise the most-imperiled class among all vertebrates [23]. This highlights the importance of preserving their biodiversity and shedding light on their phylogenetic relationships [23] by prioritizing especially threatened species as they embody significant amounts of unique evolutionary history [2].

In recent years, with the advent of molecular data, there has been a significant effort towards elucidating the evolutionary history of chondrichthyans [2,15]. However, their phylogeny is still controversial at all levels, ranging from superorders to genera [24]. A significant obstacle towards resolving their phylogenetic relationships is that several species are likely to represent complexes of several distinct species that require taxonomic resolution, for example some dogfishes, eagle rays, and stingrays [24,25,26]. Moreover, although the “Hypnosqualea” hypothesis, suggesting that batoids are derived sharks related intimately to the sawfish and angel sharks [27,28,29,30], is no longer supported [31,32,33] and it is widely believed that modern sharks (Selachii) are monophyletic, the relationships among the three main superorders (Galeomorphii, Squalomorphii and Batoidea) and the arrangement of the orders within these groups remain unsolved [15]. Notably, most of the phylogenetic studies of chondrichthyans are based on few nuclear and/or mitochondrial DNA (mtDNA) genes [2,31,34]. Individual mitochondrial markers are widely used to assess species diversity and population connectivity [35,36,37] due to the relatively simple sequencing procedures and the high rates of nucleotide substitution [38]. 

The recent blooming of Next Generation Sequencing (NGS) methodologies allowed the reliable and accurate assembly of the complete mtDNA genomes (mitogenomes) for phylogenetic analysis [15,23,39]. Currently, the complete mitogenomes have been sequenced, assembled and annotated from 82 species of sharks, 73 species of rays and 8 species of chimaeras (Table S1).

In this context, the principal objective of the present study was to sequence and characterize the complete mitogenome of nine chondrichthyans (four sharks and five rays) sampled as bycatch by commercial fisheries in Greece. The gene content, organization, codon usage and base composition were analyzed in each assembled mitogenome. The phylogenetic relations of all 172 species (including 163 mitogenomes retrieved from GenBank) were reconstructed based on Maximum Likelihood and Bayesian Inference methods that were applied to the concatenated sequences of the 13 protein coding genes of the mitogenome.

## 2. Materials and Methods

### 2.1. Tissue Sampling and DNA Extraction

Individual fin clips were obtained from four shark species (*Galeus melastomus*, *Odontaspis ferox*, *Prionace glauca* and *Squalus blainville*) and five ray species (*Bathytoshia centroura*, *Dasyatis tortonesei*, *Gymnura altavela*, *Raja undulata* and *Torpedo marmorata*) (Table 1). All specimens were incidentally caught by commercial trawlers or long-liners in the Aegean Sea, Greece and were kindly provided by fishermen. The experimental design was performed by an accredited researcher (MR: CZ03540) and was approved by the ethical committee of the Faculty of Science, Charles University, Czech Republic (UKPRF/28830/2021). Fin samples were preserved in 95% ethanol and stored at −20 °C. Total genomic DNA was extracted from approximately 25 mg of each sample using the standard protocol of the DNeasy Tissue kit (Qiagen, Chatsworth, CA, USA). The DNA concentration of each sample was estimated using NanoDrop One Spectrophotometer (Thermo Scientific, Wilmington, DE, USA). DNA fragmentation was checked with 1% agarose gel electrophoresis. Species identification was initially assessed macroscopically based on standard taxonomic features [9] and was confirmed by blasting the obtained cytochrome *c* oxidase subunit I (*COI*) gene from each assembled mitogenome to the homologous sequences deposited in GenBank, using the Basic Local Alignment Search Tool (BLAST) of the National Center for Biotechnology Information (NCBI) [40].

### 2.2. Sequencing and Mitogenome Assembly

Total DNA sequencing was performed by Novogene Bioinformatics Technology Co., Ltd. (Yuen Long, Hong Kong) in Illumina HiSeq2500 platform with 150 base pair per-end sequencing option. The reads were trimmed for adapters in Trimmomatic [41], checked for quality in FastQC [42] and mapped against a reference mitogenome from a closely related species in Geneious Prime software [43]. The reference mitogenomes are presented in Table S1. Subsequently, the mapped Illumina reads were de novo assembled with Geneious Prime [43] to reconstruct the complete mitogenome of each species. As a final step, the total DNA-seq reads were mapped to the assembled mitogenome from the same species with Geneious Prime [43] to confirm the quality of the assembly, correct potential assembly gaps, and finally close the circular molecule. The infrastructure of MetaCentrum (www.metacentrum.cz (accessed on 26 October 2020)) was used for computational resources. 

### 2.3. Annotation and Sequence Analysis 

The complete mitogenome of each species was annotated using MitoAnnotator on the MitoFish website (http://mitofish.aori.u-tokyo.ac.jp/annotation/input.html (accessed on 26 October 2020)) [44]. The programs RNAmmer (http://www.cbs.dtu.dk/services/RNAmmer/ (accessed on 26 October 2020)) [45] and tRNA scan-SE (http://lowelab.ucsc.edu/tRNAscan-SE/ (accessed on 26 October 2020)) [46] were used to confirm the ribosomal RNA (rRNA) and the transfer RNAs (tRNAs) annotation results, respectively. The secondary structures of tRNAs were predicted by MITOS (http://mitos.bioinf.uni-leipzig.de/help.py (accessed on 26 October 2020)) [47]. The control region was inspected by the program “Tandem Repeats Finder” (https://tandem.bu.edu/trf/trf.html (accessed on 26 October 2020)) [48]. The boundaries of the protein coding genes (PCGs), rRNA genes and tRNA genes were refined manually by comparison with the annotated elasmobranch mitogenomes from GenBank. The obtained complete mitogenomes were deposited in GenBank under the accession numbers MT274568–MT274576 (Table 1; Table S1).

The nucleotide composition and the A+T and G+C contents (%) were calculated for the complete mitogenome and each of the 13 PCGs per species in MEGA v5.1 [49]. The AT skews and GC skews were calculated using the following formulas: AT skew D (A-T)/(A+T), GC skew D (G-C)/(G+C) [50]. Finally, the codon usage in the mitochondrial PCGs was estimated per species by the Sequence Manipulation Suite (http://www.bioinformatics.uni-muenster.de/tools/sms2/codon_usage.html) [51]. The relative synonymous codon usage value (RSCU) of a codon, corresponding to the number of times that a codon appears in a gene in relation to the number of expected occurrences under an assumption of equal usage of synonymous codons (values less than 1 or more than 1 indicate that the codons are used less or more often than the expected) [52] was calculated in DNASP v6.12.03 [53].

### 2.4. Phylogenetic Analysis

For phylogenetic analyses, in addition to the nine assembled mitogenomes, a total of 163 complete mitochondrial genomes of sharks, rays and chimaeras were retrieved from GenBank (Table S1). The 13 PCGs were extracted, aligned with the CLUSTAL W algorithm [54], and concatenated using the software Geneious [43]. The most likely model of sequence evolution for each dataset (individual PCGs and concatenated dataset) was selected by JModelTest v2.1.7 [55], based on the Bayesian Information Criterion (Table S2). The phylogenetic relationships were initially reconstructed with the Maximum Likelihood (ML)-based approach through the online implementation of PhyML v3 provided by the Montpellier Bioinformatics Platform (http://www.atgc-montpellier.fr (accessed on 26 October 2020)) [56] with 1000 bootstrap replicates. In addition, a Bayesian Inference (BI) phylogenetic tree was constructed in MrBayes 3.2.6 [57] on the computer cluster MetaCentrum (www.metacentrum.cz (accessed on 26 October 2020)). The Bayesian Inference method was applied using the Markov Chain Monte Carlo (MCMC) algorithm from randomly generated starting trees for 5 million generations with trees sampled every 100 generations and 2 runs with 4 chains (2 heated and 2 cold). The first 25% of the trees were discarded as burn-in, and the remaining sampled trees were used to estimate the 50% majority rule consensus tree and the Bayesian posterior probabilities. For the concatenated analysis, the matrix was partitioned by gene to include gene-specific models of substitution. ML and Bayesian analyses were carried out for each individual PCG and the concatenated dataset of all 13 PCGs under the evolutionary models presented in Table S2. The Maximum Likelihood (ML) and Bayesian Inference (BI) trees were visualized and edited in FigTree v1.4.3 [58].

## 3. Results and Discussion

### 3.1. Genome Organization

The size of the assembled mitogenomes ranged from 16,682 bp in *Odontaspis ferox* to 19,472 bp in *Gymnura altavela* and was within the expected size range of the complete mtDNA sequences retrieved from GenBank (Table S1). To date the smallest mitogenome has been reported for the Chilean devil ray *Mobula tarapacana* (Philippi, 1892) [59] (15,686 bp; Accession number: MH669414) and the largest for the Pacific spookfish *Rhinochimaera pacifica* (Mitsukuri, 1895) (24,889 bp; Accession number: HM147141) [60]. The differences in the mtDNA genome size among elasmobranchs correspond mainly to the high content of tandem repeats in the control region [24].

The gene order and content of all the nine assembled mitogenomes was the typical expected for vertebrates [61]. Specifically, the mitogenome of each species contained 13 protein coding genes (PCGs), 2 ribosomal RNA (rRNA) genes, 22 transfer RNA (tRNA) genes, the control region (*D-loop*) and several small noncoding regions. The analytical description of each of the nine assembled mitogenomes is presented in Table S3. Additionally, the gene map and a short description of each assembled mitogenome are presented in Figures S1–S9.

### 3.2. Protein-Coding Genes and Codon Usage

The mitogenome of each species encoded a typical full set of 13 proteins. The majority of the PCGs were transcribed from the heavy (H) strand, except for the *ND6* gene and eight out of the 22 tRNA genes (tRNAGln, tRNAAla, tRNAAsn, tRNACys, tRNATyr, RNASer, tRNAGlu and tRNAPro), which were transcribed from the light (L) strand. The start codons were the typical ATG codon of all PCGs, regardless of the species with some exceptions: the *COI* gene was initiated by a GTG codon, *ATP6* gene was initiated by a GTG codon only in *Torpedo marmorata*, and the *ND6* gene was initiated by a CTA or TTA codon. Among the mitochondrial PCGs, the ND5 was the longest, while the ATP8 was the shortest, in all species. Most of the PCGs were terminated by a complete (TAA/TAG), while the incomplete termination codon (TA/T) was found in 5 out of the 13 PCGs. Such incomplete termination codons (TA/T) are a common phenomenon in metazoan mitogenomes and can be extended to a complete TAA termination codon through polyadenylation of the 3’-end of the mRNA, which occurs after transcription [62]. These features of the initial and stop codons are commonly observed in elasmobranchs [39,63] and are similar to the majority of the vertebrate mitochondrial PCGs [64].

The base composition and the RSCU values of the mitochondrial PCGs are presented per species in Tables S4 and S5. Each PCG and the complete mitogenome of all species were rich in the A+T content, resulting in a strong bias towards A+T rich codons in the codon usage, which appears to be a shared feature in vertebrates [61]. The most frequently used codons across species were: TTALeu (average = 4.21%), TTTPhe (average = 3.17%), CTALeu (average = 3.14%), TATTyr (average = 2.90%), CTTLeu (average = 2.79%), CCTPro (average = 2.65%), ACAThr (average = 2.59%) and ATAMet (average = 2.54%). The codons with the highest RSCU values that were found in the PCGs from the nine assembled mitogenomes were TTALeu (RSCU average = 1.76), GCCAla (average = 1.56), TCTSer (average = 1.55), TCASer (average = 1.52) and AAALys (average = 1.52) (Table S5).

### 3.3. rRNA and tRNA Genes

The mitogenome of each species contained 22 tRNA genes interspersed along the genome, the small subunit of rRNA (12S rRNA) and the large subunit of rRNA (16S rRNA). They were transcribed in the same direction on the H-strand and separated by tRNAVal. The size in base pairs (bp) of all tRNA and rRNA genes is presented in Table S2. All tRNA genes could fold into a distinctive cloverleaf secondary structure except tRNASer(AGY), which contained a simple loop without making the dihydrouridine arm, similarly to many metazoan mitogenomes [64,65] (Figure S10).

### 3.4. Noncoding Regions

The noncoding regions included the origin of light strand replication (OL), one putative control region (*D-loop*) and intergenic spacers, namely some overlapping nucleotides and gaps between PCGs or between PCGs and tRNAs (Table S2). In each assembled mitogenomes the OL region was located between the tRNAAsn and tRNACys genes and the control region was located between the tRNAPro and tRNAPhe genes. The OL region ranged in size from 32 bp in *Raja undulata* to 41 bp in *Galeus melastomus*. Moreover, the control region exhibited significant size variation among the studied species, ranging in size from 1068 bp in *Prionace glauca* to 3768 bp in *Gymnura altavela*, and was enriched in tandem repeat sequences in all species, except *Squalus blainville*. This finding confirms the fact that the control region exhibits extensive nucleotide and size polymorphism, as it has been shown in several elasmobranchs [66,67,68] and teleosts [69,70,71]. An analytical description of the tandem repeats is presented in Table S6. 

### 3.5. Phylogenetic Inference

**Figure 1:**
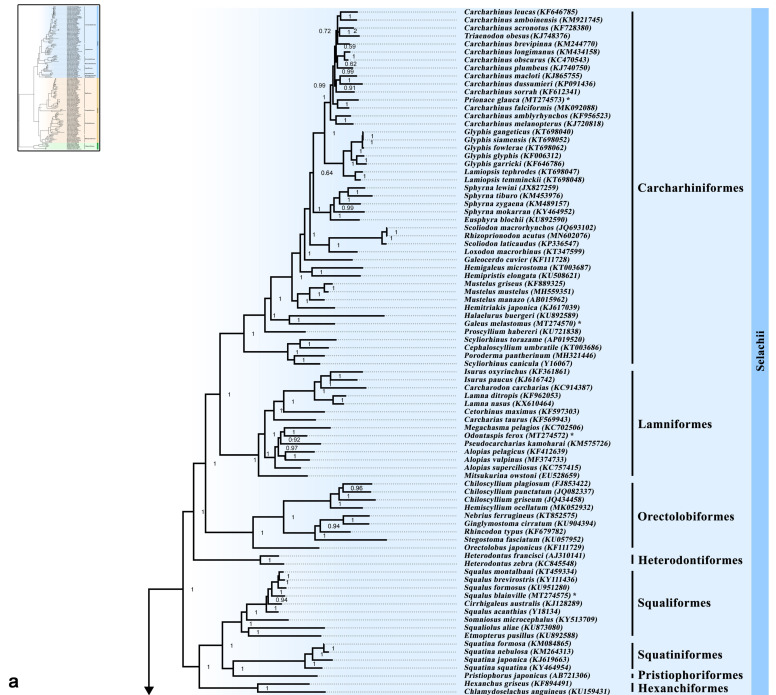

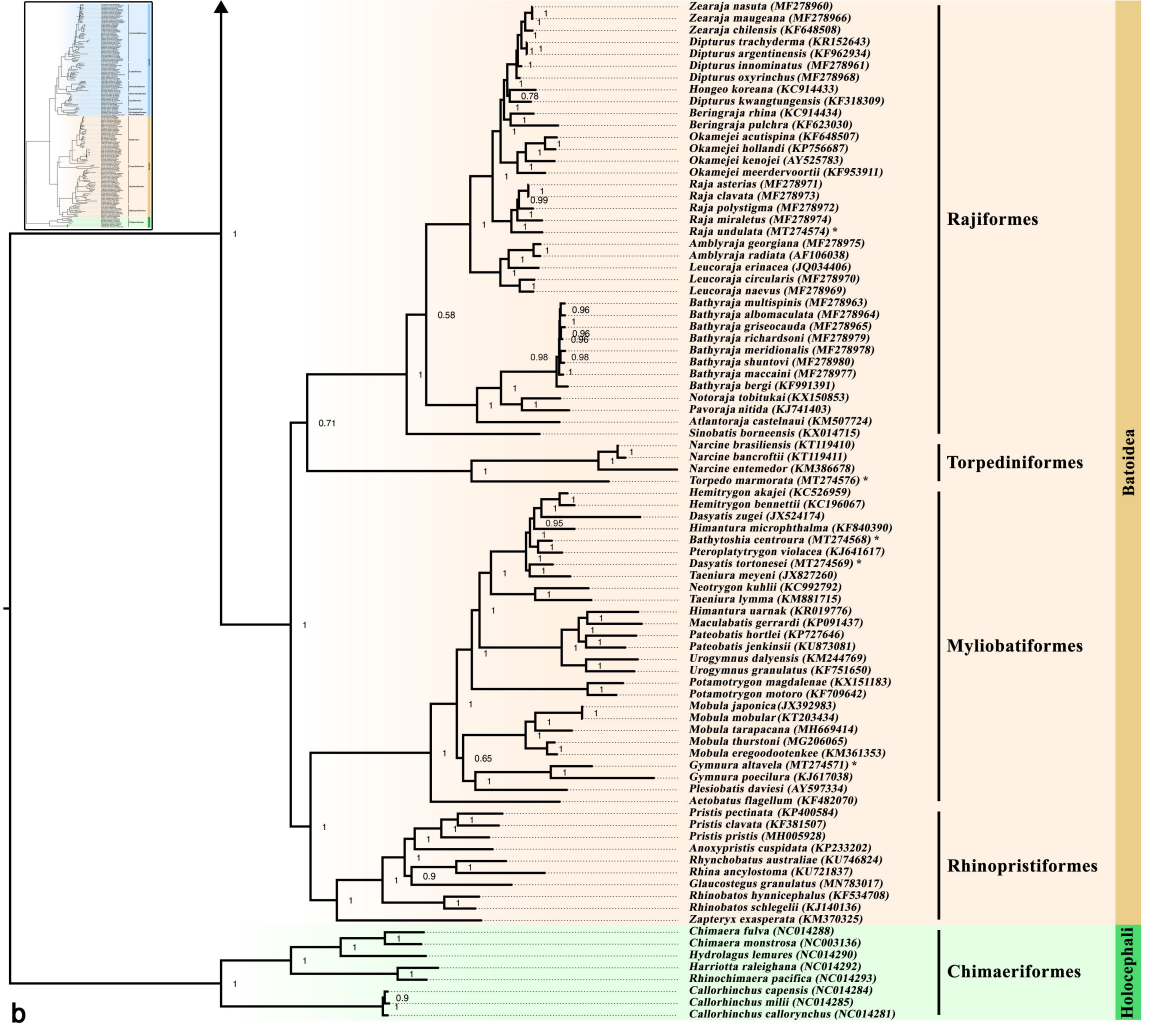
(**a**) Bayesian mitogenomic phylogeny of Selachii based on the concatenated dataset of 13 protein-coding genes. (**b**) Bayesian mitogenomic phylogeny of Batoidea and Holocephali, based on the concatenated dataset of 13 protein-coding genes. Posterior probabilities are presented next to the nodes. The complete phylogeny is illustrated in the miniphoto. Mitogenomes assembled for the first time in this study are indicated by an asterisk (*).

**Figure 2:**
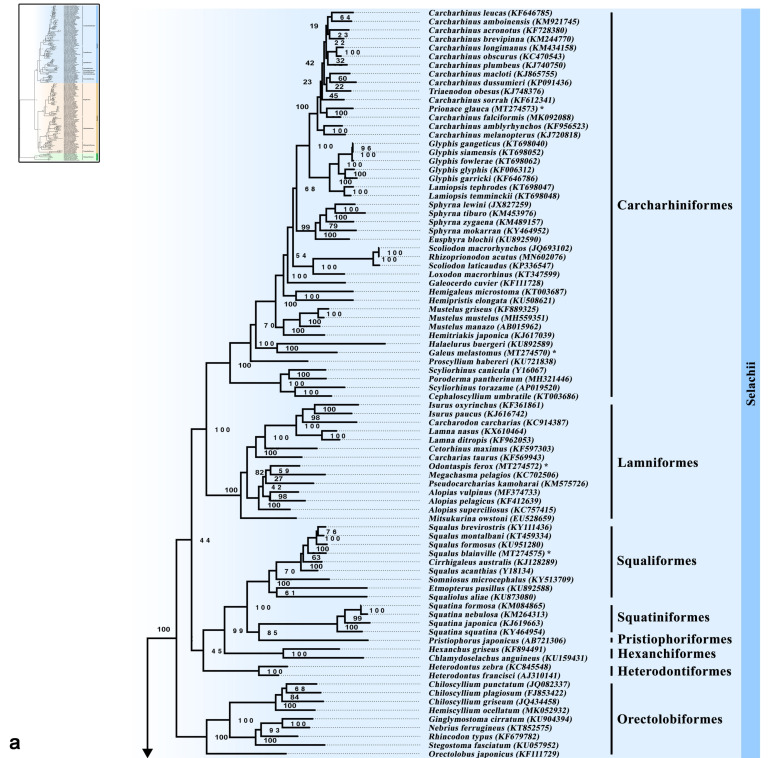

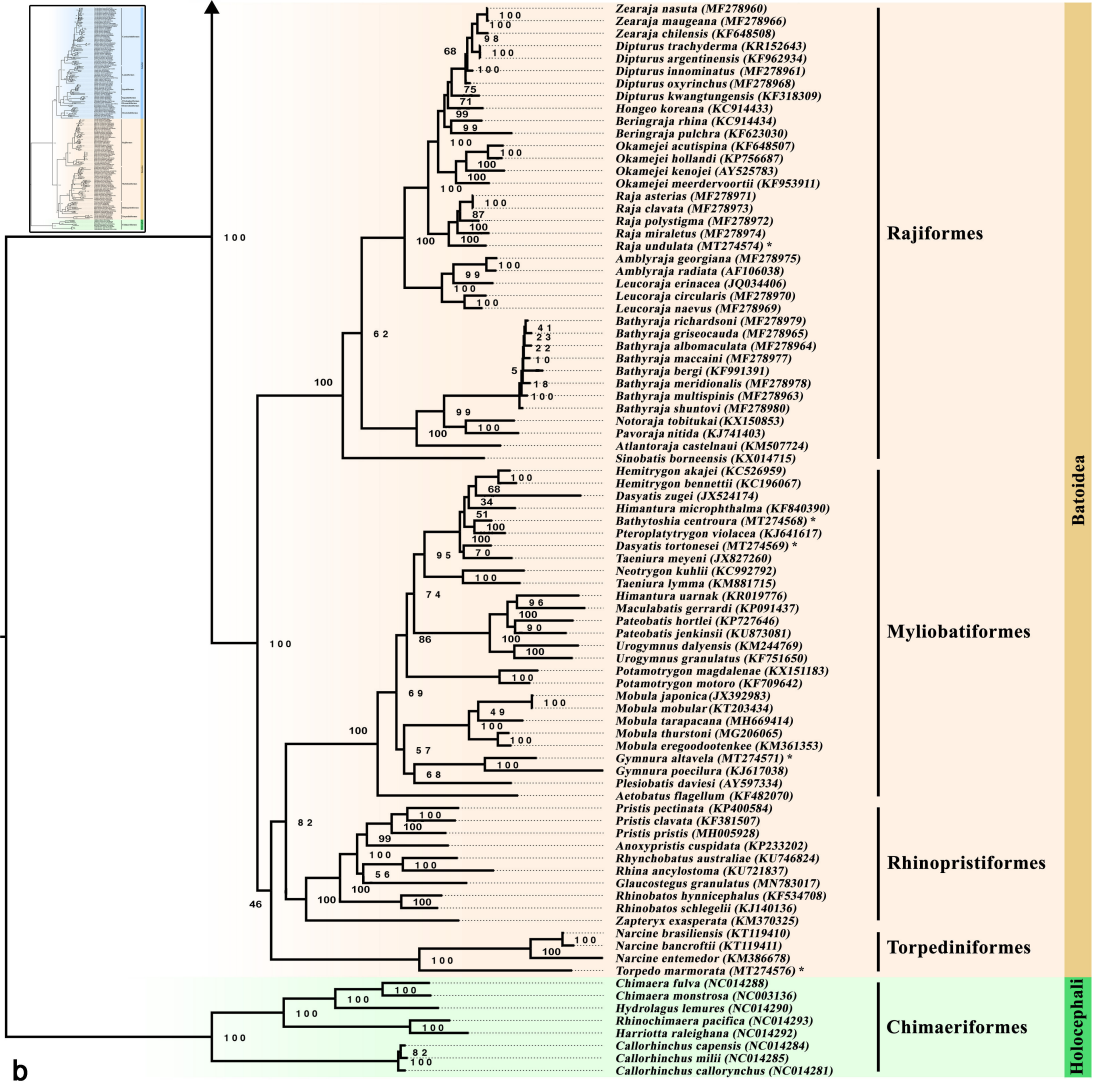
(**a**) Maximum Likelihood mitogenomic phylogeny of Selachii based on the concatenated dataset of 13 protein-coding genes. (**b**) Maximum Likelihood mitogenomic phylogeny of Batoidea and Holocephali, based on the concatenated dataset of 13 protein-coding genes. Bootstrap values are presented next to the nodes. The complete phylogeny is illustrated in the miniphoto. Mitogenomes assembled for the first time in this study are indicated by an asterisk (*).

Both ML and BI phylogenetic analyses supported the division of the class Chondrichthyes into four superorders (Galeomorphii, Squalomorphii, Batoidea and Holocephalimorpha) (Figure 1a,b and Figure 2a,b), but not the “Hypnosqualea” hypothesis, which, based on morphological traits, suggests that Batoidea is part of the shark group [27,28,29,30]. The monophyly of modern sharks has been proposed previously by Maisey [72] who morphologically identified three groups of Chondrichthyes, the first showing an orbitostylic jaw suspension (Hexanchiformes, Squaliformes, Pristiophoriformes and Squatiniformes), the second group represented by the galeomorphs (Heterodontiformes, Orectolobiformes, Lamniformes and Carcharhiniformes) and the third group with all batoids. Such classification was further supported by morphological [73,74] and recently, by molecular studies [2,8,15,31,32,33,34,75,76,77], reinforcing the scenario that the “Hypnosqualea” morphological traits could be regarded as homoplasy, due to the convergent adaptation to the benthic life [31,75]. 

The phylogenetic placement of the studied species was supported with high bootstrap and posterior probability values based on both ML and BI phylogenetic analyses (Figure 1a,b and Figure 2a,b), and notably corresponds to their reproductive mode. *Galeus melastomus* was placed within Pentanchidae (Carcharhiniformes) with oviparity as mode of reproduction [4]. *Prionace glauca* was placed within Carcharhinidae (Carcharhiniformes) with placental viviparity as mode of reproduction [4]. *Odontaspis ferox* was placed within the order Lanmniformes with oophagy (type of aplacental viviparity supported by yolk and maternal contribution) as the sole mode of reproduction [78]. *Squalus blainville* was placed within Squalidae (Squaliformes) with yolk sac viviparity (a type of aplacental viviparity where embryos feed solely on yolk) as the mode of reproduction of all Squalomorphii [4]. *Bathytoshia centroura* (Dasyatidae), *Dasyatis tortonesei* (Dasyatidae) and *Gymnura altavela* (Gymnuridae) were placed within Myliobatiformes with istotrophy (a type of aplacental viviparity supported by yolk and uterine milk) as the sole mode of reproduction [79]. Finally, *Torpedo marmorata* was placed within Torpedinidae (Torpediniformes) reproducing with istotrophy [80] and as a sister taxon to Narcinidae (Torpediniformes) reproducing with yolk sac viviparity with other maternal contribution [81]. The effect of the reproductive mode on the phylogenetic placement of elasmobranchs has also been supported by Hull et al. [82] who showed that *Mustelus mustelus* was phylogenetically closer to the placental species *Mustelus griseus*, both of which are viviparous placental, in contrast to *Mustelus manazo*, which is aplacental [83,84]. Furthermore, given that oviparity is the sole reproductive mode for all Chimaeriformes and Heterondontiformes species, and for some families of Orectolobiformes (Parascylliidae, Hemiscylliidae and Stegostomatidae), Carcharhiniformes (Scyliorhinidae and Proscyllidae) and Rajiformes (Rajidae) [4], earlier suggestions that egg-laying oviparous sharks are ancestral [81,85,86] are confirmed. 

Within Selachii, the phylogenetic analysis recovered the eight well-known shark orders divided into two distinct clades, the Squalomorphii and the Galeomorphii, being consistent with previous morphological [30,31,32,33,34,35,36,37] and molecular divisions [2,8,15,24,31,32,33,75,77]. The Squalomorphii was represented by four orders. According to the Bayesian mitogenomic phylogeny (Figure 1a), the monophyletic Hexanchiformes was placed at the most basal position and sequentially followed by Squatiniformes placed as sister taxon to Pristiophoriformes, and the monophyletic Squaliformes that was placed as a sister taxon to the Squatiniformes-Pristiophoriformes group. The proposed topology is supported with high posterior probability values and is consistent with previous molecular studies [15,24,31,32,33,34,87]. Molecular [77] and morphological studies [88] have placed Squaliformes as a sister taxon to Pristiophoriformes, while Heinicke et al. [8] suggested a sister relationship between Squaliformes and Squatiniformes. Pristiophoriformes are strongly supported as squaloid-like sharks based on morphological studies [27,28,29] by lacking the eight batoid synapomorphies [89]. Our findings also support the placement of Squatiniformes within squalimorphs, in contrast to Compagno [90] who proposed four superorders (Galeomorphii, Squalomorphii, Squatinimorphii and Batoidea). The placement of Squatiniformes and Pristiophoriformes within squalimorphs has been supported since the first extensive molecular phylogeny of Douady et al. [31], based on a fragment of the mitochondrial 12S, 16S and transfer RNA valine genes (2400 nucleotides) from over 20 elasmobranchs, until more recent studies that analyzed the complete mitogenome of more than 70 elasmobranchs [15,77]. 

Within Hexanchiformes, Hexanchinidae was placed as a sister taxon to Chlamydoselachidae. Within Squatiniformes, the basal *Squatina squatina* was grouped with *Squatina japonica* and sequentially by *Squatina nebulosa* which was placed as sister species to *Squatina formosa*. Squatinidae was placed as a sister taxon to Pristiophoridae represented by *Pristiophorus japonicus*.

Within Squaliformes, Etmopteridae was placed as a sister taxon to Dalatiidae, and both families were at the most basal position of the order. Somniosidae was placed as a sister taxon to Squalidae. Squalidae showed monophyly as in Vélez-Zuazo and Agnarsson [33] where Squalimorphii were represented by more species and families. According to Vélez-Zuazo and Agnarsson [33] Somniosidae, Dalatiidae and Etmopteridae showed paraphyly, a notion that was not confirmed in the present study, probably due to the different representation of the families. Vélez-Zuazo and Agnarsson [33] observed poor support for most of the relationships among the families of Squaliformes, while in the present study they were strongly supported. Notably, a distinct phylogenetic placement of *Squalus* species was supported by high posterior probability values, with *Squalus acanthias* recovered as the basal taxon within Squalidae. Squaliformes comprise the second most diverse order of sharks with taxonomic uncertainties within the genus *Squalus* [37]. Thus, by providing the complete mitogenome of *Squalus blainville*, the present study could support future phylogenetic studies with the scope to resolve these uncertainties. 

The Galeomorphii was represented by four monophyletic orders (Figure 1a). The basal Heterodontiformes followed by Orectolobiformes and, sequentially, by the Carcharhiniformes–Lamniformes group, a topology previously supported by morphological [28,29] and molecular studies [2,8,15,24,77]. Nevertheless, based on ML tree topology, Orectolobiformes was placed at the most basal position of a group including all of the sharks’ orders (Figure 2a). Based on BI analysis, the phylogenetic placement of Heterodontiformes as sister taxon to all other galeomorphs has already been reported [8,15,29,77,88] and opposes the close relationship between Heterodontiformes and squalimorphs suggested by Mallatt and Winchell [87] and Human et al. [32]. Moreover, the basal placement of Heterodontiformes represented by the monophyletic genus *Heterodontus*, opposes previous studies suggesting the galeomorph *Heterodontus* and the squalimorph *Chlamydoselachus* as basal neoselachians or nonneoselachian sharks [91,92]. Additionally, the Lamniformes-Carcharhiniformes grouping opposes the Lamniformes–Orectolobiformes grouping that was suggested based on sequences of the mtDNA and/or nuclear genes [31,32,33,76,77,87]. Winchell et al. [75] used nuclear major and minor rRNA subunits to relate the Lamniformes–Orectolobiformes group to the *Isurida* group that was initially proposed based on morphological similarities [93], a view not corroborated by Compagno [88]. Most of these similarities were considered by Winchell et al. [75] as homoplasy and were treated as convergent adaptations for pelagic carnivory, a view that cannot be confirmed in recent molecular phylogenetic studies [2,15,24,77].

Within Orectolobiformes, Orectolobidae was placed in a basal position related to the group formed by all other families similarly to previous studies [15,24,77]. Stegostomatidae was placed as a sister taxon to the Rhincodontidae–Ginglymostomatidae group and Hemiscylliidae was placed as a sister taxon to the Stegostomatidae–Rhincodontidae-Ginglymostomatidae group. An identical placement of the families within Orectolobiformes has been found in previous phylogenetic studies [15,33,77].

Lamniformes was represented by seven families, including the paraphyletic Alopiidae and Odontaspididae (Figure 1a). Mitsukurinidae was placed at the most basal position of all lamniforms divided into two groups: the first group formed by the basal Odontaspididae (*Carcharias taurus*), Cetorhinidae and Lamnidae and the second group formed by the basal *Alopias superciliosus* placed as a sister taxon to a group subdivided to the other representatives of the Alopiidae family and the Pseudocarcharidae-Odontaspididae–Megachasmidae group. On the contrary, Alopiidae was recovered as monophyletic and sister taxon of the Megachasmidae–Pseudocarcharidae group in Amaral et al. [77]. Our study supports the monophyly of Lamnidae as shown in previous phylogenetic studies [15,24,33,77]. The genus *Lamna* was placed as a sister taxon to the most nested taxa of *Carcharodon* and *Isurus*, a topology supported by both morphological [94] and molecular data [15,24,33,77]. Moreover, previous morphological and molecular studies [28,95] placed Mitsukuridae basal to all other Lamniformes similarly to our findings. However, in Amaral et al. [77], Mitsukurinidae was placed either as a basal sister taxon to the Alopiidae–Megachasmidae–Pseudocarcharidae group in the ML tree topology or as basal taxon to all Lamniformes in the BI tree topology.

According to the Bayesian mitogenomic phylogeny (Figure 1a), Carcharhiniformes was represented by seven families, with Carcharhinidae showing paraphyly and Scyliorhinidae placed at the most basal position of this order, similarly to previous molecular studies [15,24,33,77]. Scyliorhinidae was followed by Proscylliidae, which was sequenced by Pentanchidae and later by Triakidae and Hemigaleidae, with a grouping between Sphyrnidae and Carcharhinidae in the most nested position. The embedment of Sphyrnidae within Carcharhiniformes was also supported by Vélez-Zuazo and Agnarsson [33] based on nuclear and mitochondrial genes, and by Da Cunha et al. [15] based on complete mitogenomic analysis. Our results enhance previous morphological studies in which Scyliorhinidae represented the basal lineage followed by Triakidae and the clade formed by the derived Sphyrnidae and Carcharhinidae [88,92]. The phylogenetic placement of all families with Carcharhiniformes was supported by high posterior probability values. The only exception was in the case of Sphyrnidae (represented by *Sphyrna* and *Eusphyrna* species) that was grouped within Carcharhinidae (represented by *Carcharhinus*, *Glyphis*, *Lamniopsis*, *Prionace* and *Triaenodon* species) with 0.64 probability. A second group of Carcharhinidae was represented by *Loxodon macrorhinus* basal to the *Rhizoprionodon*-*Scoliodon* group, and it was recovered following *Galeocerdo cuvier* with high posterior probability value (1.0). Similarly, to our results, Vélez-Zuazo and Agnarsson [33] assigned *Scoliodon* as the sister genus of *Rhizoprionodon* but no *Loxodon* species was included in their analysis, while Naylor et al. [96] assigned *Scoliodon* deeply nested within the Carcharhinidae, as the sister genus of *Loxodon* in a group with Rhizoprionodon as the basal genus. Paraphyly within Carcharhinidae is commonly observed in phylogenetic studies with differences attributed to the selected genes or the number of species included in the analysis [33,77,96]. Actually, lack of monophyly has been observed in almost all of the families within Carcharhiniformes, in a variety of studies using a different combination of molecular markers, most of them from the mtDNA [32,33,97]. In the present study, most of *Carcharhinus* species were placed within Carcharhinidae with medium probability values based on BI analysis but showed polytomy in ML analysis (Figure 1a and Figure 2a). A similar pattern of polytomy within Carcharhinidae has previously been observed [15] and related to the low levels of intrinsic genetic variability of sharks [98]. 

The Batoidea were split to four monophyletic orders. Based on the BI analysis, a basal division was found between a first group recovered with medium support (0.71) and formed by Rajiformes placed as a sister taxon to Torpediniformes, and a second group recovered with strong support (1.00) and formed by Rhinopristiformes placed as a sister taxon to Myliobatiformes (Figure 1b). Rajiformes were placed as a sister taxon to Torpediniformes, similarly to the results Da Cunha [15] and Amaral et al. [77]. A low support regarding the division of Torpediniformes from other batoids was also recovered in Amaral et al. [77] where only *Narcine entemedor* was included in the analysis, but also in Gaitán-Espitia et al. [99] presenting the most complete phylogeny of Torpediniformes based on 11 PCGs and including 6 species from the genera *Torpedo*, *Typhlonarke* and *Narcine*. It is worth mentioning that based on the ML analysis, Torpediniformes was placed with low support (46) as a basal taxon to the Rhinopristiformes–Myliobatiformes group, and all these three orders were placed as a sister group to Rajiformes (Figure 2b). According to the Bayesian mitogenomic phylogeny (Figure 1b), Rajiformes was represented by three families, the basal Anacanthobatidae, Arhynchobatidae and Rajidae. Within Rajiformes, all families formed monophyletic groups with species of the genus *Bathyraja* (Arhynchobatidae) being involved in a large polytomy. Torpediniformes was represented by two families, the Torpedinidae, represented by *Torpedo marmorata*, which was placed as a sister and basal taxon to Narcinidae represented by three *Narcine* species. Rhinopristiformes was represented by four families out of which Rhinobatidae was monophyletic. *Zapteryx exasperata* was placed at the most basal position of the order and followed by a group formed by two *Rhinobatos* species. Following this, the monophyletic Pristidae was placed as a sister taxon to the Rhinobatidae–Rhinidae–Rhynchobatidae group. Myliobatiformes was represented by five families with *Aetobatus flagellum* (Myliobatidae) placed at the most basal position. The topology of all families within Myliobatiformes was highly supported with only Myliobatidae showing paraphyly. Dasyatidae was recovered as sister family to Potamotrygonidae similarly with Amaral et al. [77]. Finally, Gymnuridae was recovered as a sister taxon to Pleisiobatidae, and both families were more closely related to Myliobatidae than Dasyatidae.

## 4. Conclusions

Elasmobranchs consist one of the most diverse groups, represented by almost 1200 taxonomically valid species, and at the same time are among the most vulnerable taxa to exploitation [23]. The present study describes analytically for the first time the complete mitogenome of nine elasmobranchs, namely *Bathytoshia centroura*, *Dasyatis tortonesei*, *Galeus melastomus*, *Gymnura altavela*, *Odontaspis ferox*, *Prionace glauca*, *Raja undulata*, *Squalus blainville* and *Torpedo marmorata*. The phylogenetic placement of these species among modern elasmobranchs was highly supported based on both ML and BI phylogenetic analyses, also showing an effect of their reproductive mode. The phylogenetic tree reconstructions confirmed the monophyly of Selachii and Batoidea similarly to the most recent elasmobranch phylogenies. The tree topologies supported the division of Selachii to Squalomorphii and Galeomorphii, as well as the monophyly of the eight shark orders. Differences found regarding the phylogenetic placement at family or species level among modern elasmobranch phylogenies were mainly related to the variation in taxonomic sampling. This highlights the need to target taxonomic sampling in particular regions of the topology by prioritizing especially threatened species that embody significant amounts of unique evolutionary history [2]. As mitogenomic data from different taxa become available, evolutionary questions concerning Chondrichthyes are likely to be answered. In this respect, present findings contribute towards a more comprehensive understanding of the relationships among elasmobranchs and establishing conservation priorities, given that information about species’ evolutionary history and the status of their close relatives, can impact conservation planning, especially for those species already identified as being under some level of threat.

## Figures and Tables

**Table 1:** The complete mitogenome was assembled and annotated from four shark species (*Galeus melastomus*, *Odontaspis ferox*, *Prionace glauca* and *Squalus blainville*) and five ray species (*Bathytoshia centroura*, *Dasyatis tortonesei*, *Gymnura altavela*, *Raja undulata* and *Torpedo marmorata*). All information for the mitogenome sequence and annotation is deposited in the GenBank database under the accession numbers MT274568–MT274576. The IUCN conservation status globally and for the Mediterranean Sea is indicated per species. Abbreviations: CR, Critically Endangered; DD, Data Deficient; EN, Endangered; LC, Least Concern; NT, Near Threatened; VU, Vulnerable.

*Species*	Common Name	IUCN Conservation Status		GenBank Accession Number
		Globally	Mediterranean Sea	
*Bathytoshia centroura*	Roughtail stingray	VU	Not evaluated	MT274568
*Dasyatis tortonesei*	Tortonese’s stingray	DD	VU	MT274569
*Galeus melastomus*	Blackmouth catshark	LC	LC	MT274570
*Gymnura altavela*	Spiny butterfly ray	VU	CR	MT274571
*Odontaspis ferox*	Smalltooth sand tiger	VU	CR	MT274572
*Prionace glauca*	Blue shark	NT	CR	MT274573
*Raja undulata*	Undulate ray	EN	NT	MT274574
*Squalus blainville*	Longnose spurdog	DD	DD	MT274575
*Torpedo marmorata*	Marbled electric ray	DD	LC	MT274576

## Data Availability

The obtained complete mitogenomes were deposited in GenBank under the accession numbers MT274568–MT274576.

## Supplementary Materials

The following are available online at https://www.mdpi.com/2073-4425/12/3/324/s1, Figure S1: Representative map of the complete mitochondrial genome of *Bathytoshia centroura* (Accession Number: MT274568); Figure S2: Representative map of the complete mitochondrial genome of *Dasyatis tortonesei* (Accession Number: MT274569); Figure S3: Representative map of the complete mitochondrial genome of *Galeus melastomus* (Accession Number: MT274570); Figure S4: Representative map of the complete mitochondrial genome of *Gymnura altavela* (Accession Number: MT274571); Figure S5: Representative map of the complete mitochondrial genome of Odontaspis ferox (Accession Number: MT274572); Figure S6: Representative map of the complete mitochondrial genome of *Prionace glauca* (Accession Number: MT274573); Figure S7: Representative map of the complete mitochondrial genome of *Raja undulata* (Accession Number: MT274574); Figure S8: Representative map of the complete mitochondrial genome of *Squalus blainville* (Accession Number: MT274575); Figure S9: Representative map of the complete mitochondrial genome of *Torpedo marmorata* (Accession Number: MT274576); Figure S10: Secondary structure model of the tRNASer (AGY) gene generated in MITOS webserver of the nine elasmobranchs included in the present study; Table S1: List of the complete mitogenomes included in the present study. The assembled mitogenomes are highlighted in grey and the reference mitogenomes in bold characters. The taxonomy and IUCN status of each species is also presented; Table S2: Substitution models selected by JModelTest v2.1.7 for each PCG and overall; Table S3: Organization of the complete mitogenome of the nine elasmobranchs included in the present study; Table S4: Base composition (%) in the complete mitogenome and PCGs of the nine elasmobranchs included in the present study; Table S5: Codon usage of the PCGs of the mitogenomes of the nine elasmobranchs included in the present study; Table S6: Description of the tandem repeats found in the control region of the assembled mitogenomes.
