# Supplementary material for: Complete Mitochondrial DNA Genome of Nine Species of Sharks and Rays and Their Phylogenetic Placement among Modern Elasmobranchs
Source: Genes (Basel). 2021 Feb 24;12(3):324. doi: 10.3390/genes12030324 (PMC7995966; doi:10.3390/genes12030324)
Supplement: Supplementary file 1 [file genes-12-00324-s001.zip › Supplementary Material_Figures_S1-S10.docx]

**Supplementary Material II**

**
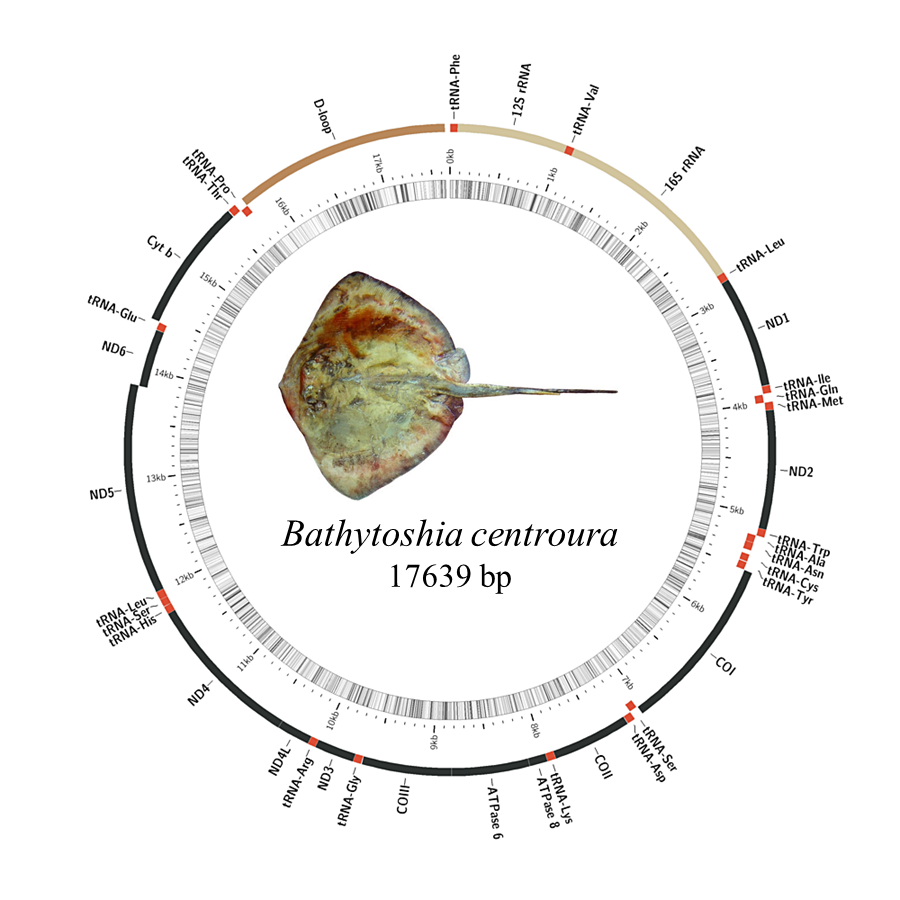
**

**Figure S1**. Representative map of the complete mitochondrial genome of *Bathytoshia centroura* (Accession Number: MT274568). Genes encoded by the heavy strand are shown outside the circle, while those encoded by the light strand are shown inside the circle.

**Description:** A total of 22 bp short overlaps were found at 4 gene junctions, with the largest one of 10 nucleotides long at the junctions of ATP8-ATP6. In addition, 51 non-coding nucleotides were observed in 16 unassigned intergenic spacers.


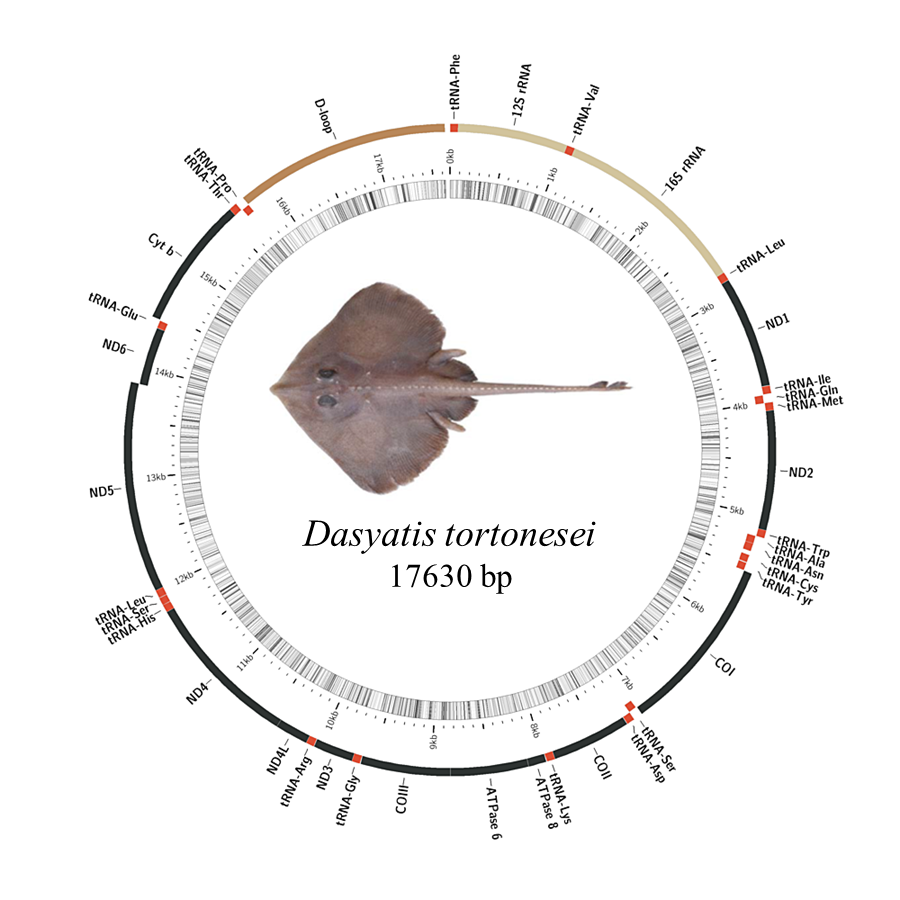


**Figure S2**. Representative map of the complete mitochondrial genome of *Dasyatis tortonesei* (Accession Number: MT274569). Genes encoded by the heavy strand are shown outside the circle, while those encoded by the light strand are shown inside the circle.

**Description:** A total of 23 bp short overlaps were found at 5 gene junctions, with the largest one of 10 nucleotides long at the junctions of ATP8-ATP6. In addition, 53 non-coding nucleotides were observed in 15 unassigned intergenic spacers.


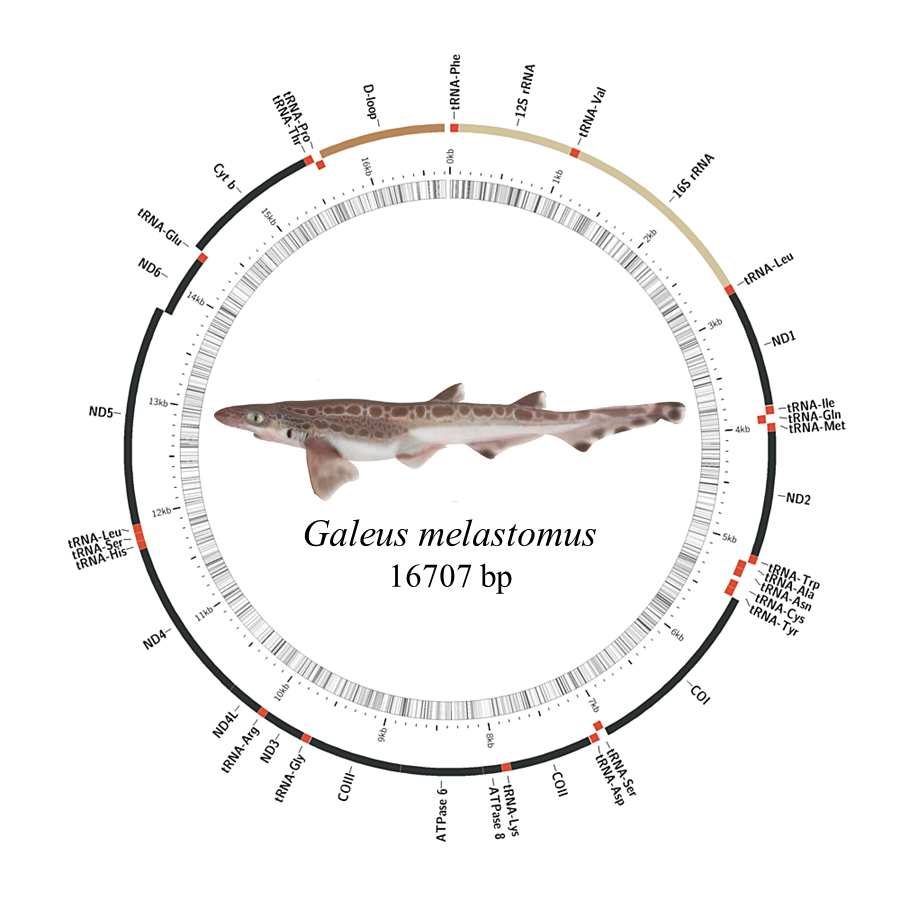


**Figure S3**. Representative map of the complete mitochondrial genome of *Galeus melastomus* (Accession Number: MT274570). Genes encoded by the heavy strand are shown outside the circle, while those encoded by the light strand are shown inside the circle.

**Description:** A total of 30 bp short overlaps were found at 6 gene junctions, with the largest one of 10 nucleotides long at the junctions of ATP8-ATP6. In addition, 22 non-coding nucleotides were observed in 10 unassigned intergenic spacers.


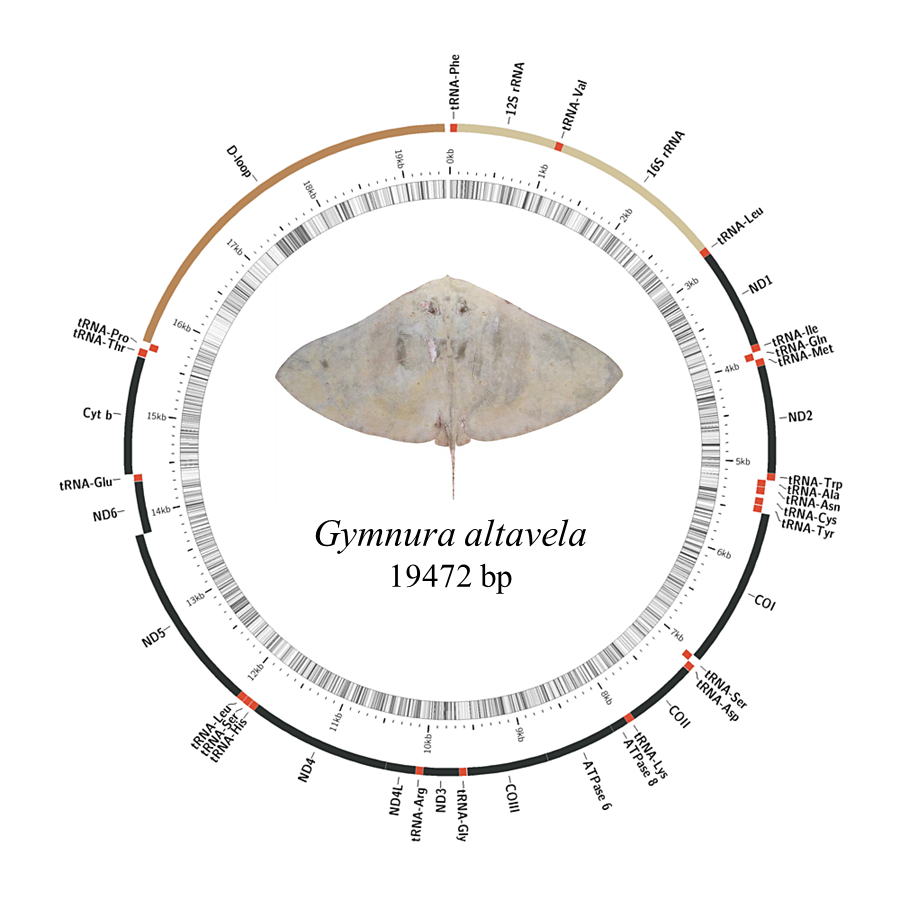


**Figure S4**. Representative map of the complete mitochondrial genome of *Gymnura altavela* (Accession Number: MT274571). Genes encoded by the heavy strand are shown outside the circle, while those encoded by the light strand are shown inside the circle.

**Description:** A total of 23 bp short overlaps were found at 5 gene junctions, with the largest one of 10 nucleotides long at the junctions of ATP8-ATP6. In addition, 45 non-coding nucleotides were observed in 15 unassigned intergenic spacers.

.
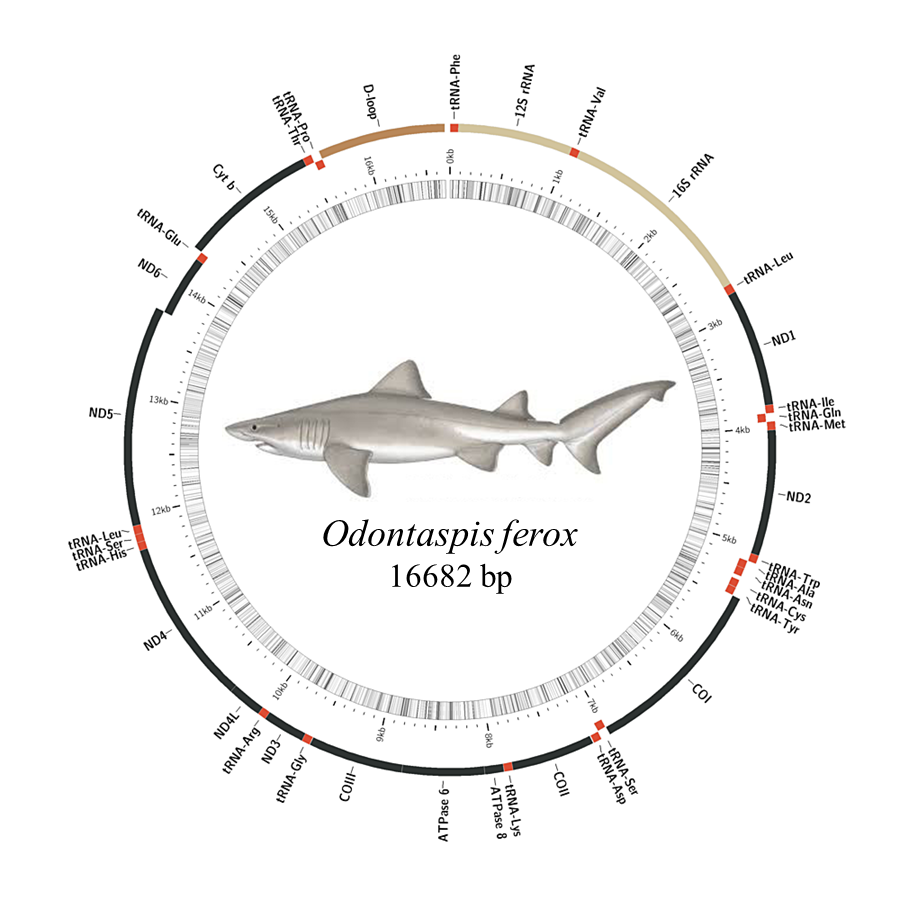


**Figure S5**. Representative map of the complete mitochondrial genome of *Odontaspis ferox* (Accession Number: MT274572). Genes encoded by the heavy strand are shown outside the circle, while those encoded by the light strand are shown inside the circle.

**Description:** A total of 24 bp short overlaps were found at 5 gene junctions, with the largest one of 10 nucleotides long at the junctions of ATP8-ATP6. In addition, 27 non-coding nucleotides were observed in 12 unassigned intergenic spacers.


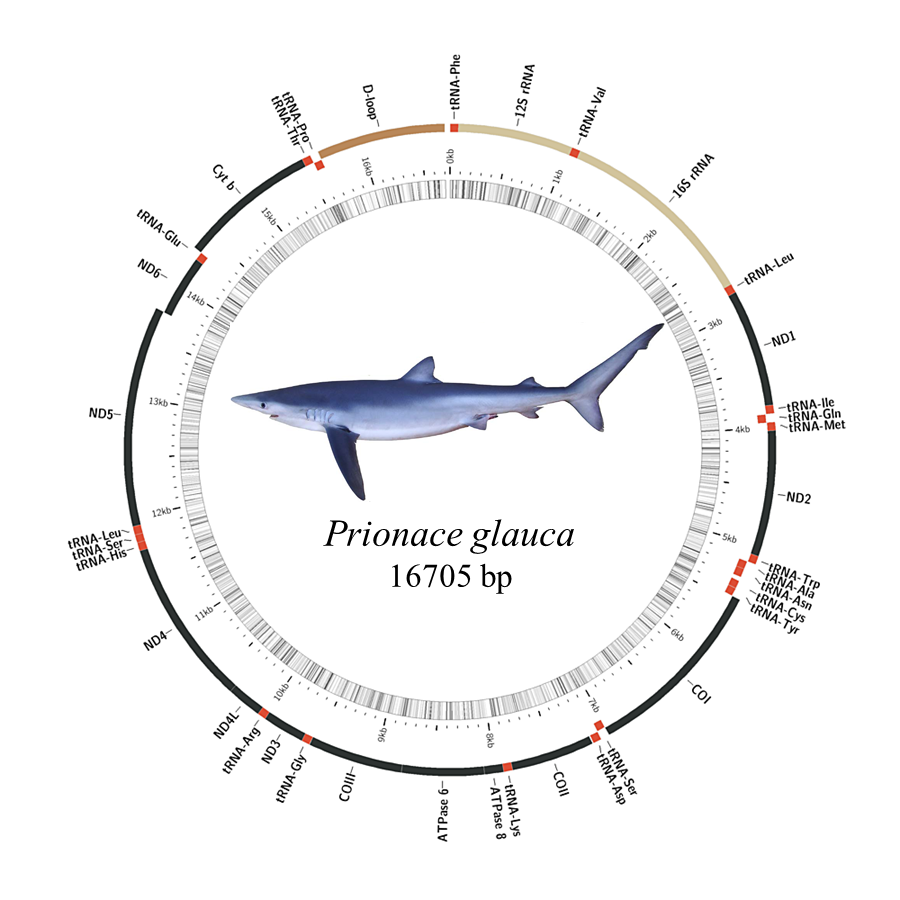


**Figure S6**. Representative map of the complete mitochondrial genome of *Prionace glauca* (Accession Number: MT274573). Genes encoded by the heavy strand are shown outside the circle, while those encoded by the light strand are shown inside the circle.

**Description:** A total of 24 bp short overlaps were found at 6 gene junctions, with the largest one of 10 nucleotides long at the junctions of ATP8-ATP6. In addition, 21 non-coding nucleotides were observed in 10 unassigned intergenic spacers.


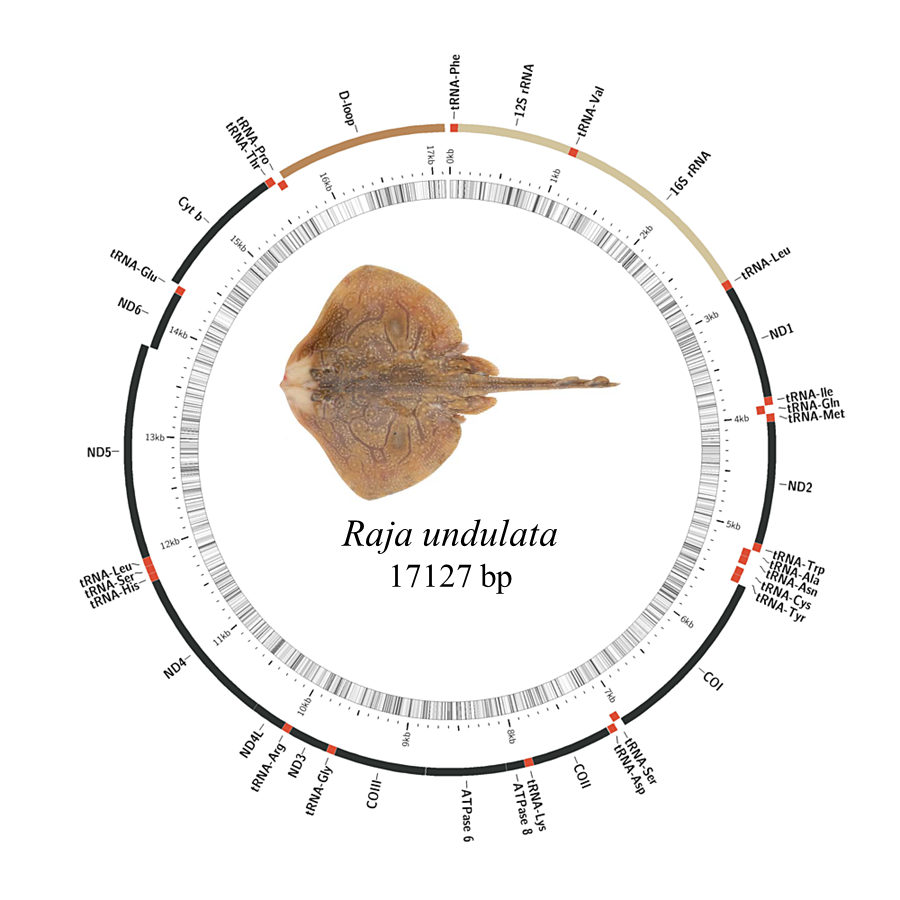


**Figure S7**. Representative map of the complete mitochondrial genome of *Raja undulata* (Accession Number: MT274574). Genes encoded by the heavy strand are shown outside the circle, while those encoded by the light strand are shown inside the circle.

**Description:** A total of 22 bp short overlaps were found at 4 gene junctions, with the largest one of 10 nucleotides long at the junctions of ATP8-ATP6. In addition, 29 non-coding nucleotides were observed in 13 unassigned intergenic spacers.


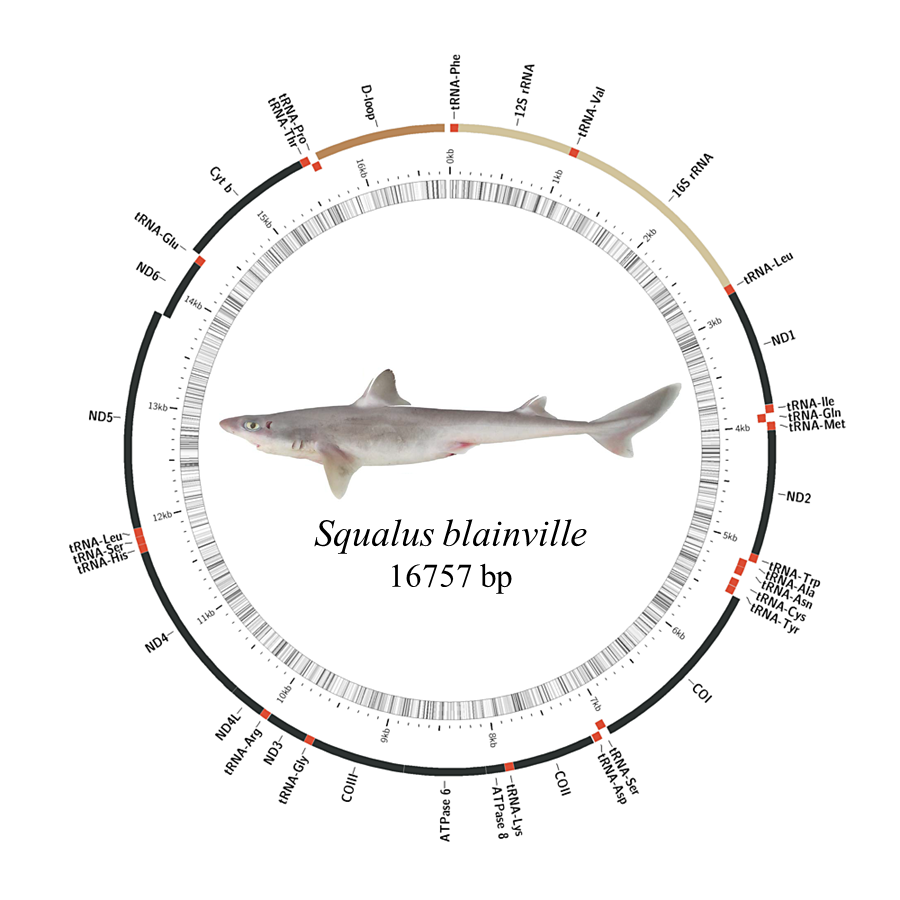


**Figure S8**. Representative map of the complete mitochondrial genome of *Squalus blainville* (Accession Number: MT274575). Genes encoded by the heavy strand are shown outside the circle, while those encoded by the light strand are shown inside the circle.

**Description:** A total of 23 bp short overlaps were found at 5 gene junctions, with the largest one of 10 nucleotides long at the junctions of ATP8-ATP6. In addition, 31 non-coding nucleotides were observed in 14 unassigned intergenic spacers.


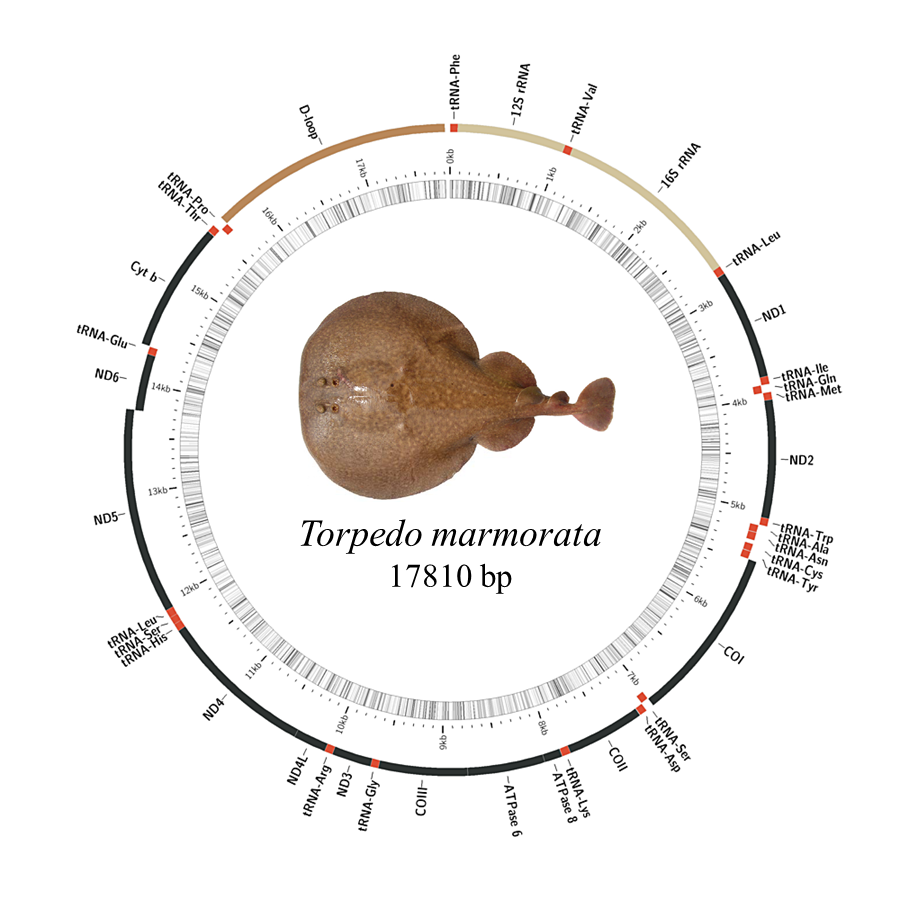


**Figure S9**. Representative map of the complete mitochondrial genome of *Torpedo marmorata* (Accession Number: MT274576). Genes encoded by the heavy strand are shown outside the circle, while those encoded by the light strand are shown inside the circle.

**Description:** A total of 23 bp short overlaps were found at 4 gene junctions, with the largest one of 10 nucleotides long at the junctions of ATP8-ATP6. In addition, 48 non-coding nucleotides were observed in 13 unassigned intergenic spacers.


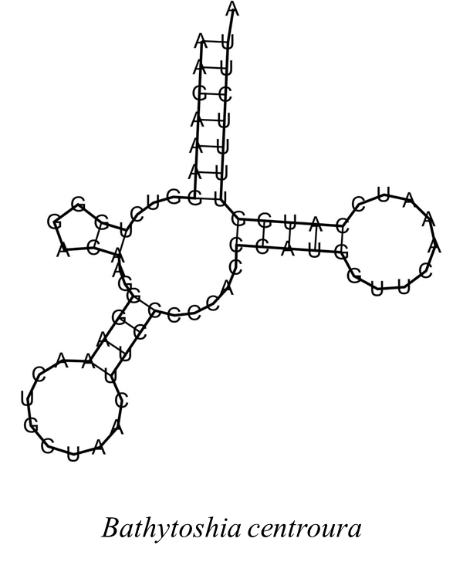

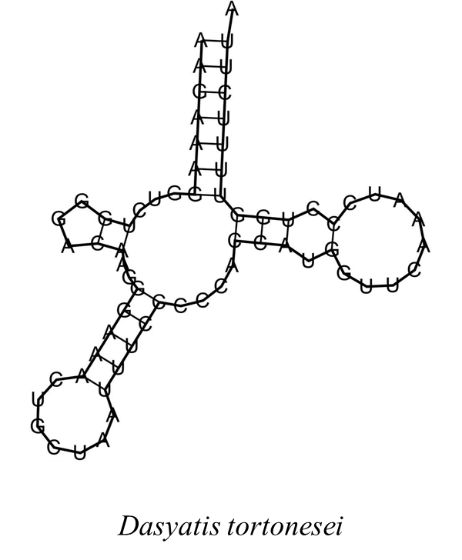

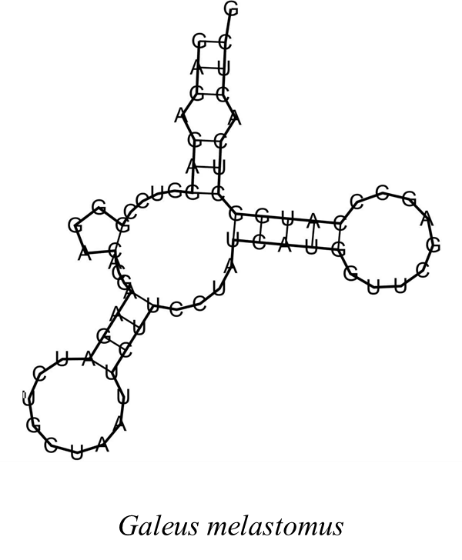


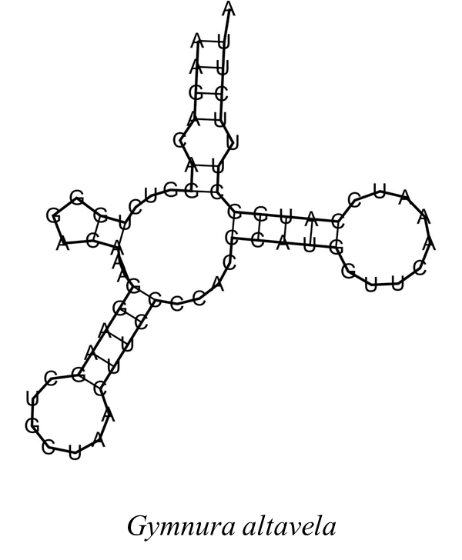

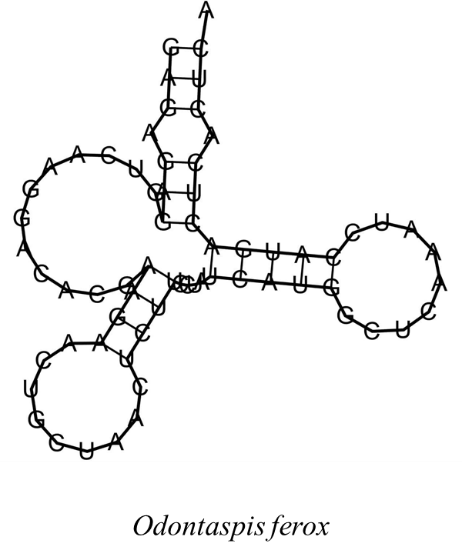

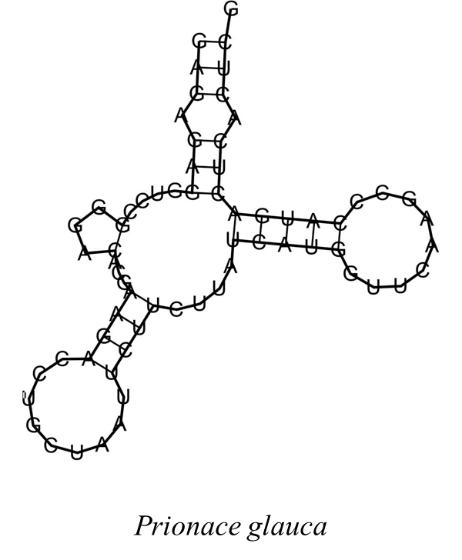


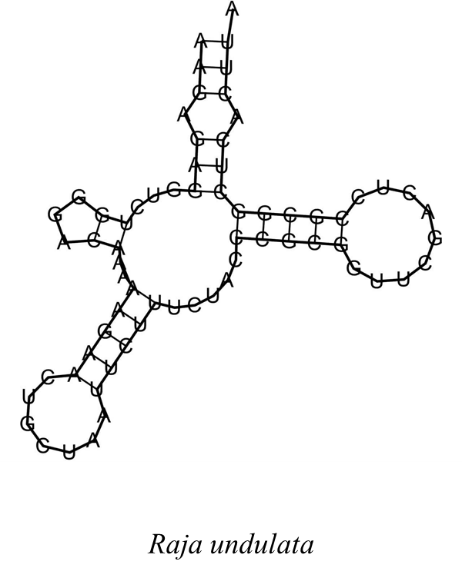

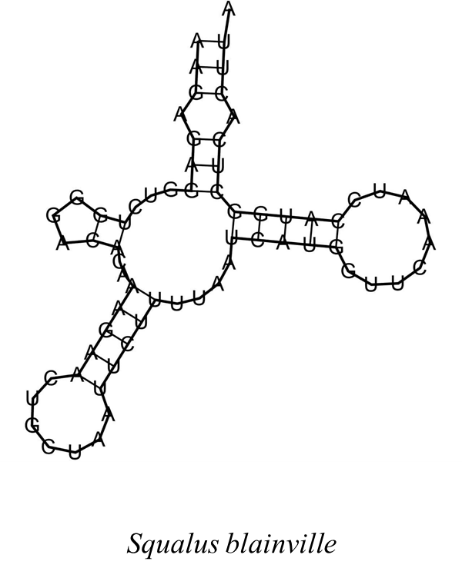

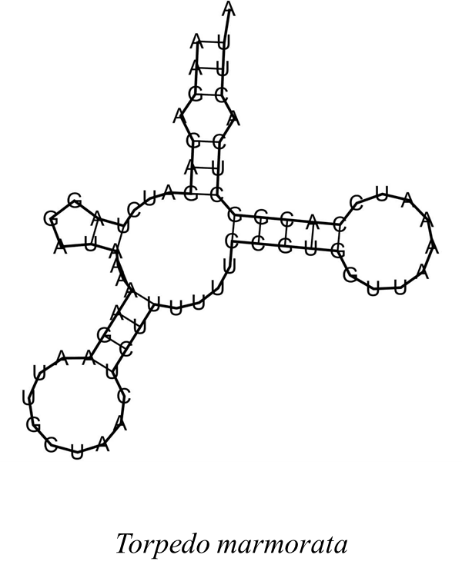


**Figure S10.** Secondary structure model of the *tRNA^Ser^* (AGY) gene generated in MITOS webserver of the nine elasmobranchs included in the present study.

**References for species’ images in Fig. S1**:

Ebert DA (2014) On Board Guide for the Identification of Pelagic Sharks and Rays of the Western Indian Ocean. FAO.

Iglésias SP (2011) Chondrichthyans from the North-eastern Atlantic and the Mediterranean (A natural classification based on collection specimens, with DNA barcodes and standardized photographs), (plates & text), Provisional version 05, 01 April 2011. 76p. <http://www.mnhn.fr/iccanam>.

https://www.fishbase.se/photos/PicturesSummary.php?StartRow=2&ID=2572&what=species&TotRec=7
